# The Role of Experiential Learning on Students’ Motivation and Classroom Engagement

**DOI:** 10.3389/fpsyg.2021.771272

**Published:** 2021-10-22

**Authors:** Yangtao Kong

**Affiliations:** ^1^School of Education, Shaanxi Normal University, Xi’an, China; ^2^Faculty of Educational Science, Shaanxi Xueqian Normal University, Xi’an, China

**Keywords:** classroom engagement, experiential learning, students’ motivation, positive psychology, education

## Abstract

Due to the birth of positive psychology in the process of education, classroom engagement has been flourished and got a remarkable role in the academic field. The other significant determining factor of success in education is motivation which is in line with classroom engagement. Moreover, based on the constructivist approach, experiential learning (EL) as a new method in education and a learner-centric pedagogy is at the center of attention, as a result of its contributions to improving the value of education which centers on developing abilities, and experiences. The current review makes an effort to consider the role of EL on students’ classroom engagement and motivation by inspecting its backgrounds and values. Subsequently, the efficacy of findings for academic experts in educational contexts is discussed.

## Introduction

It is stated that a basic causative factor in the general achievement of learners studying in higher education is learners’ engagement (Xerri et al., 2018; Derakhshan, 2021). It is extensively approved that learners who are actively participating in the learning progression and take interest in their academic education are more likely to achieve higher levels of learning (Wang et al., 2021). Therefore, higher education institutions encourage learners to use their capabilities, as well as learning opportunities and facilities that enable them to be actively engaged (Broido, 2014; Xie and Derakhshan, 2021). Moreover, students’ dissatisfaction, boredom, negative experiences, and dropping out of school are in part due to the low engagement in academic activities (Derakhshan et al., 2021). It has been demonstrated that engagement is, directly and indirectly, related to intelligence, interest, motivation, and pleasure with learning outcomes within many academic fields (Yin, 2018). Likewise, engagement is a construct that is shaped from the multifaceted relations of perceptions, feelings, and motivation which is corresponding to the progress of self-determination theory in the motivation realm (Mercer and Dörnyei, 2020). Besides, the student’s motivation is a significant factor in cultivating learning and consequently increasing the value of higher education because the more the learners are motivated, the more likely they can be successful in their activities (Derakhshan et al., 2020; Halif et al., 2020).

From a psychological point of view, motivating learners and engaging them in the classroom are closely related (Han and Wang, 2021); nevertheless, motivation consists of factors that are psychological and difficult to observe, while engagement involves behaviors that can be observed by others that it is not simple to notice and estimate learners’ motivation (Reeve, 2012). In other words, educators cannot concretely understand the fulfillment of their learners’ basic mental necessities and enthusiasm for learning (Reeve, 2012). Nonetheless, Reeve asserted that in contrast to motivation, learners’ engagement by all accounts is a phenomenon that is distinctive and can nearly be noticed. Generally, educators can impartially consider whether or not a specific learner is engaged in the class exercises, such as problem solving.

As a reaction to the traditional teaching approach that is teacher-centric (Che et al., 2021) and following the inclination to expanding interest in a more unique, participative learning atmosphere, educational organizations are orienting toward learning approaches that cultivate students’ involvement, interest, and dynamic participation. EL is a successful teaching method facilitating active learning through providing real-world experiences in which learners interact and critically evaluate course material and become involved with a topic being taught (Boggu and Sundarsingh, 2019). Based on the teaching theory of Socrates, this model relies on research-based strategies which allow learners to apply their classroom knowledge to real-life situations to foster active learning, which consequently brings about a better retrieval (Bradberry and De Maio, 2019). Indeed, engaging in daily activities, such as going to classes, completing schoolwork, and paying attention to the educator, is all indicators of classroom engagement (Woods et al., 2019). Moreover, by participating in an EL class paired with relevant academic activities, learners improve their level of inherent motivation for learning (Helle et al., 2007) and they have the opportunity to choose multiple paths to solve problems throughout the learning process by having choices and being autonomous (Svinicki and McKeachie, 2014). EL is regarded as learning by action whereby information is built by the student during the renovation of changes (Afida et al., 2012). Within EL, people become remarkably more liable for their learning which regulates a stronger connection between the learning involvement, practices, and reality (Salas et al., 2009) that are key roles in learning motivation.

To make sure that the learners gain the required knowledge and get the factual training, it is equally important to give them time to develop their ability to use their knowledge and apply those skills in real-world situations to resolve problems that are relevant to their careers (Huang and Jiang, 2020). So, it seems that they would like more hands-on training and skills development, but awkwardly, in reality, they generally just receive theoretical and academic education (Green et al., 2017). In addition, as in today’s modern world, where shrewd and high-performing people are required, motivation and engagement should be prioritized in educational institutions as they are required features in the learning setting while they are often overlooked in classrooms (Afzali and Izadpanah, 2021). Even though studies on motivation, engagement, and EL have been conducted so far; however, based on the researcher’s knowledge, just some have currently carried out systematic reviews about the issue and these studies have not been all taken together to date; therefore, concerning this gap, the current mini-review tries to take their roles into account in education.

## Classroom Engagement and Motivation

As a three-dimensional construct, classroom engagement can be classified into three types: physical, emotional, and psychological (Rangvid, 2018). However, it is not always easy to tell whether a learner is engaged because observable indicators are not always accurate. Even those who display signs of curiosity or interest in a subject or who seem engaged may not acquire knowledge about it. Others may also be learning despite not displaying any signs of physical engagement (Winsett et al., 2016).

As an important component of success and wellbeing, motivation encourages self-awareness in individuals by inspiring them (Gelona, 2011). Besides, it is a power that manages, encourages, and promotes goal-oriented behavior, which is not only crucial to the process of learning but also essential to educational achievement (Kosgeroglu et al., 2009). It appears that classroom motivation is influenced by at least five factors: the learner, the educator, the course content, the teaching method, and the learning environment (D’Souza and Maheshwari, 2010).

## Experiential Learning

EL, developed by Kolb in 1984, is a paradigm for resolving the contradiction between how information is gathered and how it is used. It is focused on learning through experience and evaluating learners in line with their previous experiences (Sternberg and Zhang, 2014). The paradigm highlights the importance of learners’ participation in all learning processes and tackles the idea of how experience contributes to learning (Zhai et al., 2017). EL is a method of teaching that allows learners to learn while “Do, Reflect, and Think and Apply” (Butler et al., 2019, p. 12). Students take part in a tangible experience (Do), replicate that experience and other evidence (Reflect), cultivate theories in line with experiences and information (Think), and articulate an assumption or elucidate a problem (Apply). It is a strong instrument for bringing about positive modifications in academic education which allow learners to apply what they have learned in school to real-world problems (Guo et al., 2016). This way of learning entails giving learners more authority and responsibility, as well as involving them directly in their learning process within the learning atmosphere. Furthermore, it encourages learners to be flexible learners, incorporate all possible ways of learning into full-cycle learning, and bring about effective skills and meta-learning abilities (Kolb and Kolb, 2017).

## Implications and Future Directions

This review focused on the importance of EL and its contributions to classroom engagement and motivation. Since experiential education tends to engage a wider range of participants who can have an impact on the organization, employees, educators, leaders, and future colleagues, it is critical to maintain its positive, welcoming atmosphere. The importance of EL lies in its ability to facilitate connections between undergraduate education and professional experience (Earnest et al., 2016), so improving the connection between the university and the world of work (Friedman and Goldbaum, 2016).

The positive effect of EL has actual implications for teachers who are thinking of implementing this method in their classes; indeed, they can guarantee their learners’ success by providing them with the knowledge required in performing the task as following the experiential theory, knowledge is built through converting practice into understanding. Based on the literature review, the conventional role of the teacher shifts from knowledge provider to a mediator of experience through well-known systematic processes. Likewise, teachers should encourage learners by providing information, suggestion, and also relevant experiences for learning to build a learning milieu where they can be engaged in positive but challenging learning activities that facilitate learners’ interaction with learning materials (Anwar and Qadir, 2017) and illustrates their interest and motivation toward being a member of the learning progression. By learners’ dynamic participation in experiential activities, the teacher can trigger their ability to retain knowledge that leads to their intrinsic motivation and interest in the course material (Zelechoski et al., 2017).

The present review is significant for the learners as it allows them to model the appropriate behavior and procedures in real-life situations by putting the theory into practice. Indeed, this method helps learners think further than memorization to evaluate and use knowledge, reflecting on how learning can be best applied to real-world situations (Zelechoski et al., 2017). In the context of EL, students often find activities challenging and time-consuming which necessitates working in a group, performing work outside of the classroom, learning and integrating subject content to make decisions, adapt procedures, compare, and contrast various resources of information to detect a difficulty at one hand and implement that information on the other hand to form a product that aims to solve the issue. Participation, interaction, and application are fundamental characteristics of EL. During the process, it is possible to be in touch with the environment and to be exposed to extremely flexible processes. In this way, education takes place on all dimensions which cover not only the cognitive but also the affective and behavioral dimensions to encompass the whole person. Learners enthusiastically participate in mental, emotional, and social interactions during the learning procedure within EL (Voukelatou, 2019). In addition, learners are encouraged to think logically, find solutions, and take appropriate action in relevant situations. This kind of instruction not only provides opportunities for discussion and clarification of concepts and knowledge, but also provides feedback, review, and transfer of knowledge and abilities to new contexts.

Moreover, for materials developers and syllabus designers to truly start addressing the learners’ motivation and engagement, they could embrace some interesting and challenging activities because when they can find themselves successful in comprehending the issue and being able to apply their information to solve it; they are not only more interested to engage in the mental processes required for obtaining knowledge but also more motivated and eager to learn. More studies can be conducted to investigate the effect of EL within different fields of the study courses with a control group design to carry out between-group comparisons. Besides, qualitative research is recommended to scrutinize the kinds of EL activities which make a more considerable effect on the EFL learners’ motivation and success and even their achievement.

## Author Contributions

The author confirms being the sole contributor of this work and has approved it for publication.

## Funding

This study was funded by the Projects of National Philosophy Social Science Fund, PRC (17CRK008), and the Projects of Philosophy and Social Science Fund of Shaanxi Province, PRC (2018Q11).

## Conflict of Interest

The author declares that the research was conducted in the absence of any commercial or financial relationships that could be construed as a potential conflict of interest.

## Publisher’s Note

All claims expressed in this article are solely those of the authors and do not necessarily represent those of their affiliated organizations, or those of the publisher, the editors and the reviewers. Any product that may be evaluated in this article, or claim that may be made by its manufacturer, is not guaranteed or endorsed by the publisher.
